# Identifying Locations with Possible Undetected Imported Severe Acute Respiratory Syndrome Coronavirus 2 Cases by Using Importation Predictions

**DOI:** 10.3201/eid2607.200250

**Published:** 2020-07

**Authors:** Pablo Martinez De Salazar, René Niehus, Aimee Taylor, Caroline O’Flaherty Buckee, Marc Lipsitch

**Affiliations:** Harvard T.H. Chan School of Public Health, Boston, Massachusetts, USA

**Keywords:** outbreak, severe acute respiratory syndrome coronavirus 2, coronavirus, respiratory infections, SARS-CoV-2, 2019 novel coronavirus disease, COVID-19, travelers health, pneumonia, viruses, zoonoses

## Abstract

Cases of severe acute respiratory syndrome coronavirus 2 (SARS-CoV-2) infection exported from mainland China could lead to self-sustained outbreaks in other countries. By February 2020, several countries were reporting imported SARS-CoV-2 cases. To contain the virus, early detection of imported SARS-CoV-2 cases is critical. We used air travel volume estimates from Wuhan, China, to international destinations and a generalized linear regression model to identify locations that could have undetected imported cases. Our model can be adjusted to account for exportation of cases from other locations as the virus spreads and more information on importations and transmission becomes available. Early detection and appropriate control measures can reduce the risk for transmission in all locations.

A novel coronavirus, later named severe acute respiratory syndrome coronavirus 2 (SARS-CoV-2), was identified in December 2019 in the city of Wuhan, capital of Hubei Province, China, where cases were first confirmed (*1*). During December 2019–February 2020, the number of confirmed cases increased drastically. Model estimates suggested that >75,000 persons were infected by January 25, 2020, and the epidemic had a doubling time of ≈6 days (*2*). By the end of January 2020, travel restrictions were implemented for Wuhan and neighboring cities. Nonetheless, the virus spread from Wuhan to other cities in China and outside the country. By February 4, 2020, a total of 23 locations outside mainland China reported cases, 22 of which reported imported cases; Spain reported a case caused by secondary transmission (*3*).

Most cases imported to other locations have been linked to recent travel history from China (*3*), suggesting that air travel plays a major role in exportation of cases to locations outside of China. To prevent other cities and countries from becoming epicenters of the SARS-CoV-2 epidemic, substantial targeted public health interventions are required to detect cases and control local spread of the virus. We collected estimates of air travel volume from Wuhan to the 27 most connected locations outside of China from a total of 194 international destinations. We then identified 49 locations with high surveillance capacity according to the Global Health Security (GHS) Index (*4*). We assumed these locations would have relatively high proficiency in detecting SARS-CoV-2 and reporting confirmed imported cases, which we refer to as imported-and-reported cases. We ran a generalized linear regression model on this subset of locations; based on this model fit, we generated predictions for all international locations. Using these predictions, we identified locations that might not be detecting imported cases.

## Methods

 To identify locations reporting fewer than predicted imported SARS-CoV-2 infected cases, we fit a model to data from 49 locations outside mainland China that had a score of >49.2/100 (the 75th quantile) of the GHS Index’s category 2 (Early Detection and Reporting of Epidemics of Potential International Concern) (*4*). Among these, 17 had high travel connectivity to Wuhan and 32 had low connectivity to Wuhan (S. Lai et al., unpub. data, https://doi.org/10.1101/2020.02.04.20020479). We considered locations to be countries without taking any position on territorial claims. We performed a Poisson regression by using the cumulative number of imported-and-reported SARS-CoV-2 cases in these 49 countries and the estimated number of daily airline passengers from the Wuhan airport. We then compared predictions from this model with imported-and-reported cases across 194 locations from the GHS Index, excluding China as the epicenter of the outbreak.

The model requires data on imported-and-reported cases of SARS-CoV-2 infection, daily air travel volume, and surveillance capacity. We obtained data on imported-and-reported cases aggregated by destination from the World Health Organization technical report issued February 4, 2020 (*3*). We assumed a case count of 0 for locations not listed. We used February 4 as the cutoff for cumulative imported-and-reported case counts because exported cases from Hubei Province dropped rapidly after this date (*3*), likely because of travel restrictions for the province implement on January 23. We defined imported-and-reported cases as those with known travel history from China; of those, 83% had a travel history from Hubei Province and 17% traveled from unknown locations in China (*3*). We excluded reported cases likely caused by transmission outside of China or cases in which the transmission source was still under investigation (*3*). In addition, we excluded Hong Kong, Macau, and Taiwan from our model because locally transmitted and imported cases were not disaggregated in these locations. 

We obtained data on daily air travel from a network-based modeling study (S. Lai et al., unpub. data, https://doi.org/10.1101/2020.02.04.20020479) that reported monthly air travel volume estimates for the 27 locations outside mainland China that are most connected to Wuhan. These estimates were calculated from International Air Travel Association data from February 2018, which includes direct and indirect flight itineraries from Wuhan. For these 27 locations, estimated air travel volumes are >6 passengers/day. We assumed that travel volumes for locations not among the most connected are censored by a detection limit. We used a common method of dealing with censored data from environmental sampling (*5*), or metabolomics (*6*), and set the daily air travel volume to half the minimum value. Therefore, we used 3 passengers/day for estimated travel volumes for the 167 locations from the GHS Index not listed by Lai et al. We tested the robustness of our results by using a set of alternative values of 0.1, 1, and 6 passengers/day for the censored data. 

As noted, we defined high surveillance locations as those with a GHS Index for category 2 above the 75th quantile (*4*). We assessed among the high and low surveillance locations the numbers of zero and of nonzero imported-and-reported case counts (Table).

**Table:** Surveillance capacity of locations with and without imported-and-reported cases of severe acute respiratory syndrome coronavirus 2, 2020*

Surveillance capacity	No. locations		Total
	0 cases	>1 case	
High	35	14	49
Low	138	7	145
Total	173	21	194

*Aggregated case counts collected during January 20–February 4, 2020. Surveillance capacity reported by category 2, Early Detection and Reporting of Epidemics of Potential International Concern, of the Global Health Security Index (*3*). High surveillance capacity is defined as 1st quartile ranking of the GHS Index; low surveillance capacity are locations below the 1st quartile ranking of the GHS.

**Figure 3:**
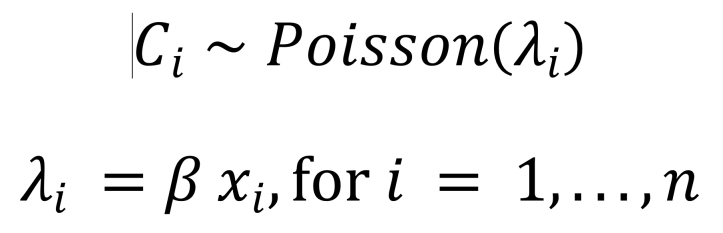
Formula.

For our model, we assumed that the cumulative imported-and-reported case counts across 49 high surveillance locations follow a Poisson distribution from the beginning of the epidemic until February 4, 2020. Then the expected case count is linearly proportional to the daily air travel volume in the following formula (Figure 3) where *i* denotes location, *C_i_* denotes the imported-and-reported case count in a location, *λ_i_* denotes the expected case count in a location, β denotes the regression coefficient, and *x_i_* denotes the daily air travel volume from Wuhan to a location. The Poisson model assumes cases are independent and that the variance is equal to the expected case count. Imported-and-reported cases likely meet the independence assumption because the value excludes cases with local transmission. We also checked the robustness of our results by using an over dispersed model with a negative binomial likelihood. We computed the p value of the overdispersion parameter as shown in Gelman and Hill (*7*).

**Figure 1:**
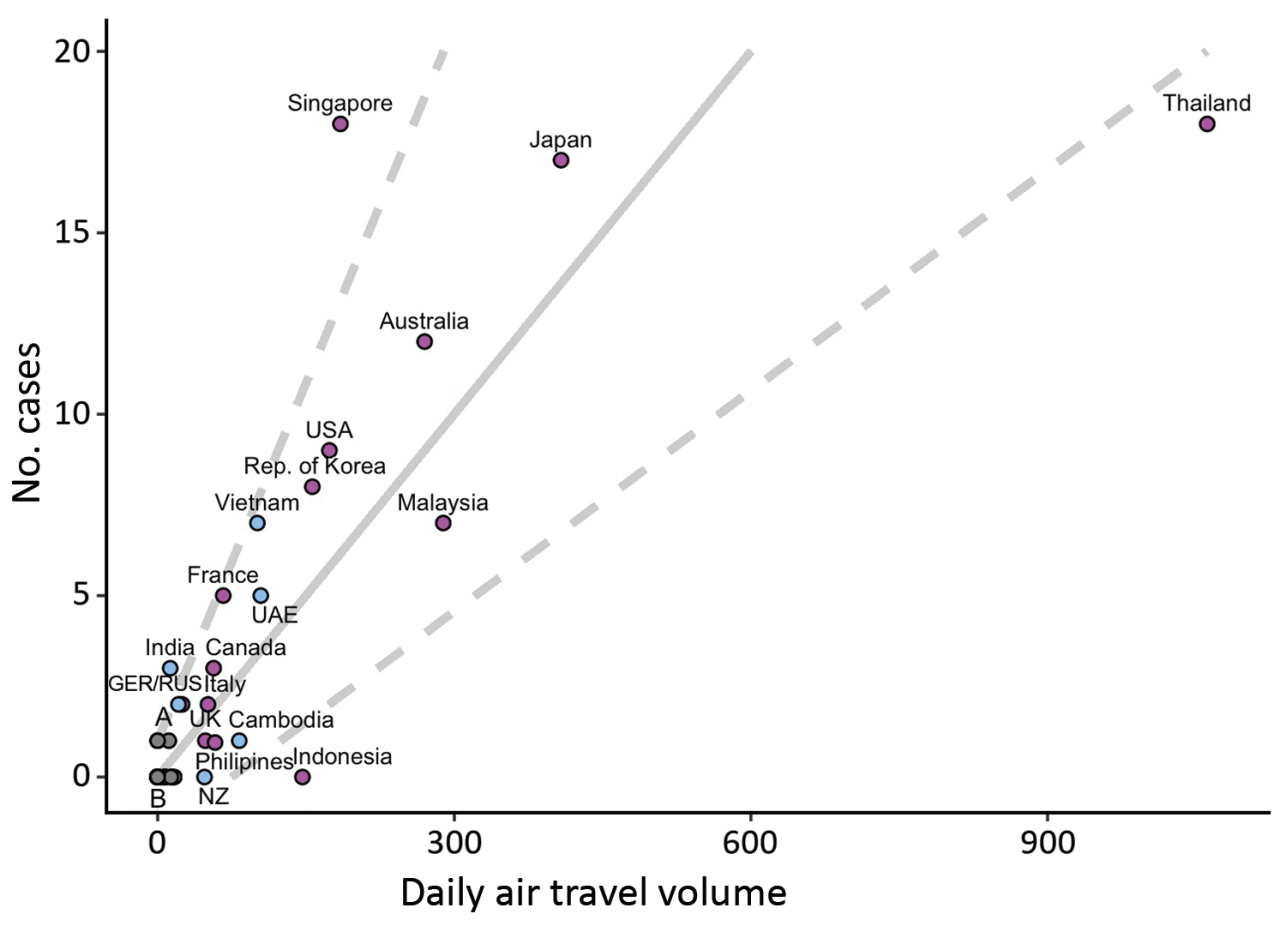
Plot showing imported-and-reported cases of severe acute respiratory syndrome coronavirus 2 (SARS-CoV-2) against air travel volume (no. persons/day) from Wuhan, China. No. cases refers to possible imported-and-reported SARS-CoV-2 cases. Solid line indicates the expected imported-and-reported case counts for locations based on the model fit to high surveillance locations (slope = 3.3 cases/100 passengers; p<0.001) . Dashed lines represent for the same model the smoothed 95% prediction interval bounds. Purple dots indicate locations with high surveillance capacity according to category 2 of the Global Health Security Index. Cluster A is composed of Nepal, Sri Lanka, Finland, and Sweden, locations with 1 imported-and-reported case and air travel volume of <20 passengers per day. Cluster B is composed of 161 locations with no imported-and-reported cases and estimated air travel <10 passengers per day. GER, Germany; NZ, New Zealand; RUS, Russia; UAE, United Arab Emirates; UK, United Kingdom; USA, United States of America.

We used R version 3.6.1 (https://www.r-project.org) to compute , the maximum likelihood estimate of β*,* and the expected imported-and-reported case count given high surveillance (Figure 1). We also computed the 95% prediction interval (PI) bounds under this model of high surveillance for all 194 locations of daily air travel volume (Figure 1). First, we generated a bootstrapped dataset by sampling *n* locations with replacement among high surveillance locations. Then, we reestimated β by using the bootstrapped dataset. Finally, we simulated imported-and-reported case counts for all 194 locations under our model by using the estimate of β from the bootstrapped dataset. We repeated the 3 steps 50,000 times to generate 50,000 simulated imported-and-reported case counts for each of the locations computed to the lower and upper PI bounds (PI 2.5%–97.5%). We smoothed the 95% PI bounds by using ggplot2 in R (*8*). We fit the imported-and-reported case counts of the 49 high surveillance locations to the model and plotted these alongside 145 locations with low surveillance capacity (Figure 1). We noted some overlap between high and low surveillance locations (Figure 1).

**Figure 2:**
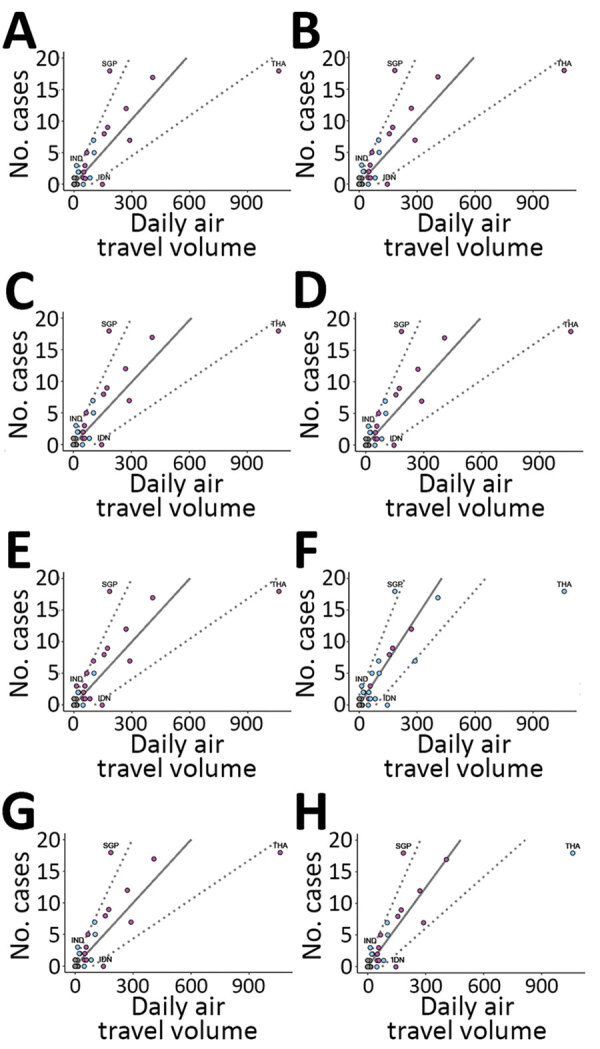
Analyses of imported-and-reported cases and daily air travel volume using a model to predict locations with potentially undetected cases of severe acute respiratory virus 2 (SARS-CoV-2). Air travel volume measured in number of persons/day. No. cases refers to possible undetected imported SARS-CoV-2 cases. Solid line shows the expected imported-and-reported case counts based on our model fitted to high surveillance locations, indicated by purple dots. Dashed lines indicate the 95% prediction interval bounds smoothed for all locations, including those with low surveillance capacity, indicated by light blue dots. A–C) Regressions that set the daily air travel volume for locations not listed by S. Lai et al. (unpub. data, https://doi.org/10.1101/2020.02.04.20020479): A) air travel volume set to 0.1 passenger/day; B) volume set to 1 passengers/day; C) volume set to 6 passengers/day. D) Regression removing locations not listed by Lai et al. before fitting. E) Regression defining high surveillance locations by using a more lenient Global Health Security (GHS) Index criterion (50th quantile) for category 2, Early Detection and Reporting of Epidemics of Potential International Concern, to define high surveillance locations. F) A more stringent GHS Index criterion (95th quantile) to define high surveillance locations. G) Regression using a negative binomial likelihood and estimated dispersion parameter of 1.27 (p = 0.097). H) Regression excluding Thailand from the model fit. Across all 8 regression analyses, Singapore lies above the 95% PI and Thailand and Indonesia lie below. India remained above 95% PI for all regressions, except when we used a more stringent GHS Index criterion (panel F). IDN, Indonesia; IND, India; SGP, Singapore; THA, Thailand.

To assess the robustness of our results we ran 8 additional regression analyses by implementing a series of changes to the analysis. These changes included the following: setting the daily air travel volume to 0.1, 1, or 6 passengers/day for locations not listed by Lai et al. (unpub. data, https://doi.org/10.1101/2020.02.04.20020479) (Figure 2, panels A–C); removing all locations not listed by Lai et al. before fitting (Figure 2, panel D); defining high surveillance locations by using a more lenient GHS Index criterion, 50th quantile (Figure 2, panel E), and a more stringent criterion, 95th quantile (Figure 2, panel F); excluding Thailand from the model because it is a high-leverage point (Figure 2, panel G); and using an overdispersed Poisson likelihood (i.e., a negative-binomial likelihood) (Figure 2, panel H). We provide code for these analyses on GitHub (https://github.com/c2-d2/cov19flightimport).

## Results

We found that daily air travel volume positively correlates with imported-and-reported case counts of SARS-CoV-2 infection among high surveillance locations (Figure 1). We noted that increasing flight volume by 31 passengers/day is associated with 1 additional expected imported-and-reported case. In addition, Singapore and India lie above the 95% PI in our model; Singapore had 12 more imported-and-reported cases (95% PI 6–17 cases) than expected and India had 3 (95% PI 1–3 cases) more than expected. Thailand has a relatively high air travel volume compared with other locations, but it lies below the 95% PI, reporting 16 (95% PI 1–40 cases) fewer imported-and-reported cases than expected under the model. Indonesia lies below the PI and has no imported-and-reported cases, but the expected case count is 5 (95% PI 1–10 cases) in our model. Across all 8 robustness regression analyses, we consistently observed that Singapore lies above the 95% PI and Thailand and Indonesia lie below (Figure 2). India remains above the 95% PI in all robustness analyses except when we used the more stringent GHS Index, 95th quantile, for fitting; then India lies on the upper bound of the 95% PI (Figure 2, panel F).

## Discussion

We aimed to identify locations with likely undetected or underdetected imported cases of SARS-CoV-2 by fitting a model to the case counts in locations with high surveillance capacity and Wuhan-to-location air travel volumes. Our model can be adjusted to account for exportation of cases from locations other than Wuhan as the outbreak develops and more information on importations and self-sustained transmission becomes available. One key advantage of this model is that it does not rely on estimates of incidence or prevalence in the epicenter of the outbreak. Also, we intentionally used a simple generalized linear model. The linearity of the expected case count means that we have only 1 regression coefficient in the model and no extra parameters. The Poisson likelihood then captures the many 0-counts observed for less highly connected locations but also describes the slope between case-count and flight data among more connected locations. We believe this model provides the most parsimonious phenomenologic description of the data.

According to our model, locations above the 95% PI of imported-and-reported cases could have higher case-detection capacity. Locations below the 95% PI might have undetected cases because of expected imported-and-reported case counts under high surveillance. Underdetection of cases could increase the international spread of the outbreak because the transmission chain could be lost, reducing opportunities to deploy case-based control strategies. We recommend rapid strengthening of outbreak surveillance and control efforts in locations below the 95% PI lower bound, particularly Indonesia, to curb potential local transmission. Early detection of cases and implantation of appropriate control measures can reduce the risk for self-sustained transmission in all locations.
